# Impact of treatment escalation on rehospitalization among patients with pulmonary arterial hypertension

**DOI:** 10.1038/s41598-025-90975-4

**Published:** 2025-04-10

**Authors:** Jeremy A. Mazurek, Hayley D. Germack, Marjolaine Gauthier-Loiselle, Ambika Satija, Ameur M. Manceur, Sherry Shi, Martin Cloutier, Patrick Lefebvre, Sumeet Panjabi

**Affiliations:** 1 University of Pennsylvania, Philadelphia, USA; 2 Medical Affairs, Johnson and Johnson Innovative Medicines, Titusville, USA; 3 Analysis Group, Inc., Montreal, Canada; 4 Analysis Group, Inc., Boston, USA

**Keywords:** Pulmonary arterial hypertension (PAH), Economic burden, Insurance claims, Hospitalization, Combination therapy, Health care economics, Hypertension

## Abstract

Pulmonary arterial hypertension (PAH) poses a substantial burden, including hospitalizations. This study assessed the impact of treatment escalation on rehospitalization. The Komodo Research Data (10/2015–03/2022) was used to identify adults with ≥ 1 PAH-related hospitalization (*index:* first hospitalization). Patients on monotherapy pre-index were assigned to the *Escalation-to-combination cohort* (treatment added ≤ 90 days post-index) or the *Monotherapy cohort* (no treatment change ≤ 90 days post-index). A sensitivity analysis was conducted among all patients who were treated pre-index. Entropy balancing was used to create cohorts with similar characteristics. All-cause hospitalizations per-patient-per-month (PPPM) during ≤ 12 months post-index were compared across balanced cohorts. A total of 203 and 1252 patients were included in the Escalation-to-combination and Monotherapy cohorts, respectively (mean age: 61 vs. 62 years; 67% vs. 68% female); most received PDE5i monotherapy pre-index (65.3% vs. 75.9%). Post-index, 84.5% of the Escalation-to-combination cohort increased to dual therapy, most commonly PDE5i + ERA (39.4%) and PDE5i + PPA (24.7%). Rehospitalization was lower in the Escalation-to-combination than Monotherapy cohort (incidence rate ratio [95% confidence interval]: 0.69 [0.55–0.90]; p < 0.001); the sensitivity analysis yielded similar results. Treatment escalation was associated with a lower rehospitalization rate, suggesting that earlier escalation and broader use of combination therapy may reduce PAH burden.

## Introduction

Pulmonary arterial hypertension (PAH) is a rare and potentially fatal subgroup of pulmonary hypertension characterized by abnormally high pressure in the pulmonary arteries and increased pulmonary vascular resistance, with a prevalence estimated at 12.4 cases per million adult inhabitants in the United States (US)^1,2^. PAH is associated with a substantial economic burden due to frequent hospitalizations^3–15^, which may in turn reflect the severe disease and poor clinical outcomes among many patients with PAH^16–18^. Multiple factors might explain the poor outcomes in PAH clinical practice, including the common phenomenon of delayed diagnosis^19,20^. Another potentially important factor is the underutilization of combination therapies, despite high-quality evidence of their efficacy and guideline recommendations^14,21^.

Over the past two decades, the treatment paradigm for patients with PAH has evolved from monotherapy to combination therapy as the latter has usually been associated with improved outcomes^22–28^. Large, randomized controlled trials have demonstrated that combination regimens improve progression-free survival for patients with PAH^23,24^. Further, meta-analyses have found that combination therapies significantly reduce the risk of clinical worsening in PAH compared to monotherapy^25,26^. Based on this evidence, the 2022 European Society of Cardiology (ESC) and European Respiratory Society (ERS) guidelines have recommended at diagnosis the use of oral double combination therapy with phosphodiesterase type 5 inhibitors (PDE5is) and endothelin receptor antagonists (ERAs) for low-to-intermediate risk treatment-naïve patients without cardiopulmonary comorbidities and triple combination therapy including with intravenous (i.v.) or subcutaneous (s.c.) prostacyclin pathway agents (PPAs) for high-risk patients without cardiopulmonary comorbidities (if adding i.v. or s.c. prostacyclin analogs is unfeasible, adding selexipag or switching from PDE5i to riociguat may also be considered)^21^. However, according to the COMPERA registry study, only 42% of patients without cardiopulmonary comorbidities reached a low risk profile with initial PAH treatment^29^. For patients who do not remain low risk after initial combination therapy with a PDE5i and ERA, the recommended treatment at follow-up is escalation to triple therapy by adding another oral agent (i.e., selexipag) or a parenteral PPA (i.e., i.v. epoprostenol or s.c. treprostinil); alternatively, switching from PDE5i to riociguat may be considered^21^. Among patients with cardiopulmonary comorbidities, the guidelines recommend initial monotherapy with a PDE5i or ERA, while the addition of another PAH medication among intermediate-to-high risk patients may be considered on an individual basis at follow-up, as treatment escalation is supported by evidence from the AMBITION and GRIPHON trials^21,23,30^. While initial dual combination therapy is associated with clinical benefits in PAH^31^, a prior study found that approximately 25% of patients who received initial dual therapy escalated to triple combination therapy after a median follow-up of 17 months^32^.

Although the benefits of combination therapy are well documented, there is a persistent gap between PAH clinical practice and current guidelines^4,7,14,33^. Indeed, clinicians continue to heavily rely on monotherapy for the treatment of all patients with PAH, suggesting that the real-world management of PAH is currently suboptimal^7,14,33^. Most notably, the majority of patients with PAH continue to be treated with monotherapy even following hospitalization^14^. Given that hospitalization is an important prognostic indicator of PAH disease progression and mortality^3–13,34^, the lack of subsequent treatment change is particularly concerning. Additionally, prior real-world studies have shown that the risk of rehospitalization is high among patients with PAH, which may signal clinical worsening and further add to the economic burden of disease^6,34–36^. Therefore, it is critical to devise strategies aimed at reducing PAH disease progression, which leads to costly and burdensome hospital stays, including readmissions. Treatment escalation (i.e., the addition of a new PAH treatment to an existing regimen) should ideally take place before disease progression and/or hospitalization, as clinical trial data suggest that proactive use of combination therapy could be beneficial for preventing such events^23,24,37^. Nonetheless, treatment escalation following hospitalization could help to address this issue by reducing the risk of rehospitalization relative to no treatment change, particularly among patients treated with monotherapy. However, there is currently a lack of real-world evidence to support this hypothesis. To address this, the current study aimed to quantify the impact of treatment escalation on rehospitalization following PAH-related hospitalization among treated patients with PAH.

## Methods

### Data source

This study used Komodo Research Data (KRD) spanning from October 1, 2015 to March 31, 2022. KRD is a de-identified database including patients from all US regions, across Medicaid, commercial, and Medicare Advantage insurers^38^. For this study, the closed subset was used, which comprises medical and pharmacy claims, as well as patient enrollment in their health care plan. Data were de-identified and comply with the patient requirements of the Health Insurance Portability and Accountability Act (HIPAA) of 1996; therefore, no review by an institutional review board was required per Title 45 of CFR, Part 46.101(b)(4).

### Study design

**Fig. 1:**
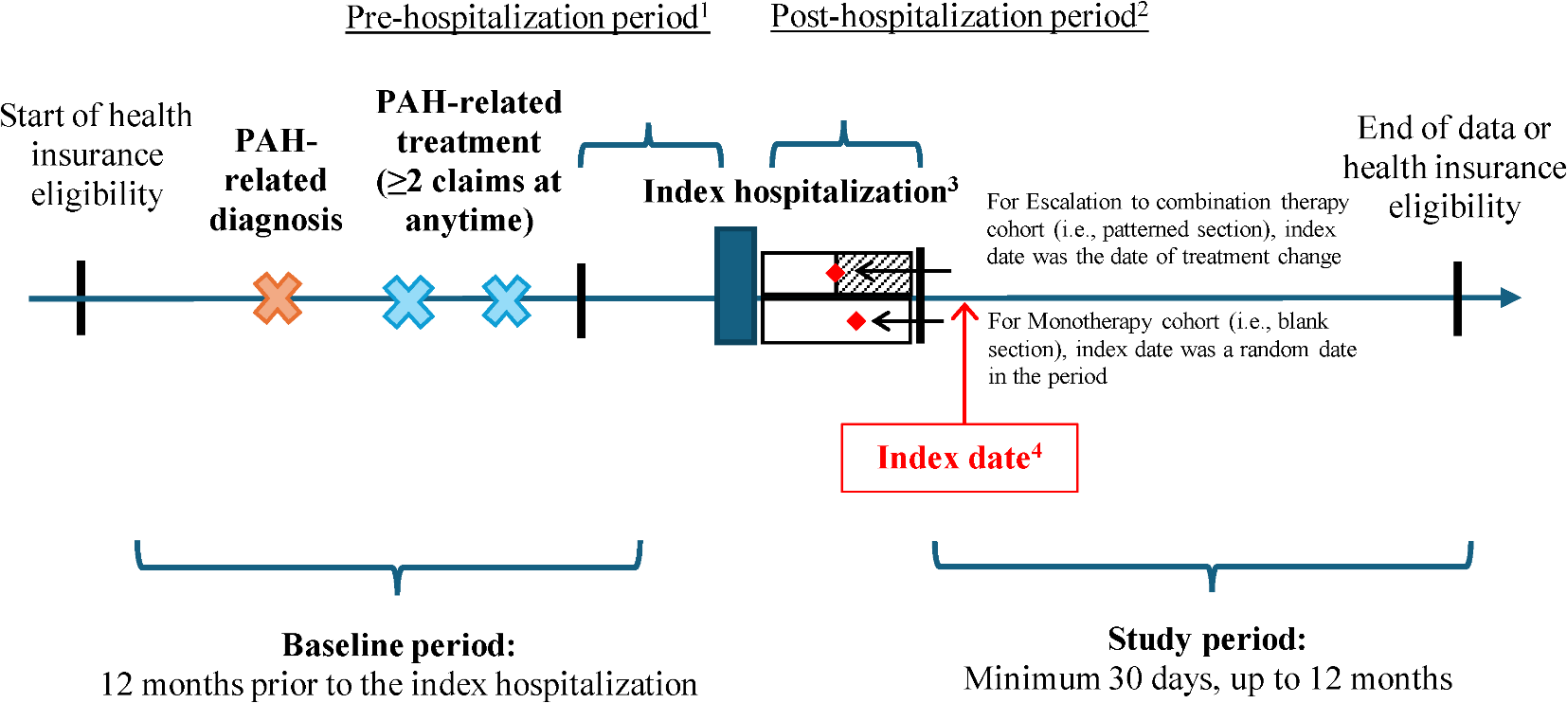
Study design.* ICD-10-CM* International Classification of Diseases, 10th Revision, Clinical Modification, *PAH* Pulmonary arterial hypertension. Note: [1] The pre-hospitalization period was defined as the 90-day period before the index hospitalization admission date and was used to identify treatment(s) before hospitalization: only patients on monotherapy were included. [2] The post-hospitalization period was defined as the 90-day period after the index hospitalization discharge date and was used to identify treatment(s) after hospitalization. [3] The first PAH-related diagnosis (ICD-10-CM: I27.0, I27.20, I27.21, I27.89) or right heart failure (ICD-10-CM: I50.81) hospitalization after the first PAH-related diagnosis was defined as the index hospitalization. [4] The index date was defined as the first fill/procedure for the last new class added in the post-hospitalization period for those who escalated or switched, or a randomly selected date in the post-hospitalization period for those who de-escalated or did not change treatment.

A retrospective cohort study design was used to address the study objectives (Fig. 1). Adults with ≥ 1 PAH-related hospitalization post-diagnosis were identified, with the first PAH-related hospitalization defined as the *index hospitalization*. Treatment escalation was assessed using an approach adapted from a prior study by Ogbomo et al.^14^ Specifically, the treatment(s) received within the 90 days prior to the index hospitalization admission date (i.e., *pre-hospitalization period*) were assessed, along with treatment(s) received during the 90 days after the discharge date of the index hospitalization (i.e., *post-hospitalization period*). In the main analysis, only patients treated with monotherapy pre-index were included. Patients who remained treated on the same drug class as monotherapy post-hospitalization were assigned to the *Monotherapy cohort* whereas those who added one or more new drug classes in the post- vs. pre-hospitalization period were assigned to the *Escalation to combination therapy cohort*. Additionally, a sensitivity analysis was conducted among all treated patients (i.e., monotherapy or combination therapy pre-index). For this sensitivity analysis, patients treated with a greater number of drug classes in the post- vs. pre-hospitalization period were classified as having had treatment escalation (*Escalation cohort*) whereas those who remained on the same drug class were classified as having had no treatment change (*No treatment change cohort*).

PAH drug classes included PDE5i, ERA, PPA, and soluble guanylate cyclase stimulator (sGCS). For the purpose of cohort classification, parenteral (i.e., subcutaneous and intravenous) PPAs were considered as separate drug class from oral or inhaled PPAs with the exception of parenteral selexipag, which was considered as the same treatment class as oral selexipag as it is not intended for chronic use. An intent-to-treat approach was used to evaluate study outcomes^39^, meaning that any change in treatment after the 90-day post-hospitalization period was not considered when defining study cohorts.

The *baseline period* was defined as the 12-month period before the admission date for the index hospitalization. The *index date* was defined as either (1) the date of the first prescription fill/procedure claim for the last new class added within the 90-day post-hospitalization period for those who escalated, or (2) a randomly selected date in the 90-day post-hospitalization period for those who did not change their treatment. The *study period* was defined as the period spanning from the index date until the earliest of 12 months, the end of continuous health insurance eligibility, or the end of data availability. A minimum study period of 30 days was required to ensure sufficient follow-up to observe rehospitalization and avoid extreme outliers (e.g., due to very short follow-up of a few days).

### Sample selection and cohorts

Eligible patients were required to have ≥ 2 documented PAH-related diagnoses (International Classification of Disease, 10th Revision, Clinical Modification [ICD-10-CM]: I27.0, I27.20, I27.21, I27.89) in any setting on distinct dates at any time. Patients were also required to have ≥ 2 prescription fills for a PAH treatment at any time and ≥ 1 PAH-related hospitalization after the date of the first PAH-related diagnosis, defined as a hospitalization with a PAH-related primary diagnosis^14^, or right heart failure primary diagnosis (ICD-10-CM: I50.81) as this is reportedly the leading cause of hospitalization in patients with PAH)^40^. Other eligibility requirements included being ≥ 18 years of age as of the admission date of the index hospitalization, having ≥ 1 PAH treatment during the 90-day period before the admission date of the index hospitalization, and continuous insurance eligibility (both medical and pharmacy) for ≥ 12 months prior to the index hospitalization admission date until the latest of ≥ 90 days post-discharge date or ≥ 30 days following the index date. Finally, patients were excluded if they had a documented diagnosis for chronic thromboembolic pulmonary hypertension (CTEPH; ICD-10-CM: I27.24) or a CTEPH-related procedure (i.e., pulmonary endarterectomy or balloon pulmonary angioplasty) at any time or a documented diagnosis for pregnancy/labor (ICD-10-CM: O00.xx-O9A.xx) during the baseline and study periods. Importantly, in the main analysis, only patients treated with monotherapy pre-index were included; a sensitivity analysis was conducted among all treated patients (i.e., monotherapy or combination therapy pre-index).

### Measures, outcomes, and statistical analyses

Patient characteristics and PAH treatments received in the pre- and post-hospitalization periods were descriptively reported. Entropy balancing was used to reweight patients in the Escalation to combination therapy cohort such that they have similar characteristics to those in the Monotherapy cohort^41,42^. Specifically, cohorts were balanced based on the following patient characteristics: age, gender, region, admission year of index hospitalization, time from first PAH-related treatment to the index hospitalization admission date, time between discharge and index date, and the following variables measured during the baseline period (i.e., 12 months pre-hospitalization): Quan-Charlson comorbidity index (CCI)^43^, right heart catheterization (RHC) procedure, receiving anticoagulants, receiving antihypertensives, chronic obstructive pulmonary disorder, anemia, connective tissue disease/rheumatic disease, hypothyroidism, number of treatment classes, and ≥ 1 inpatient stay. Additionally, cohorts were balanced with respect to a simplified claims-based risk score, a previously validated algorithm that predicts unsatisfactory response to treatment based on time since treatment initiation and PAH-related outpatient visits, pulmonologist visits, all-cause hospitalizations in the prior year, as well as the presence of a diagnosis of dyspnea, pulmonary fibrosis, and chest pain^44^. To limit the extent to which extreme values could drive the results, weights were capped at the 5th and 95th percentile. Standardized differences (STD) were used to define balance; values varying from 0.10 to 0.25 are typically judged as reasonable cutoffs^45^. In the sensitivity analysis, patients in the Escalation cohort were reweighted such that they have similar characteristics to those in the No treatment change cohort as described above.

All-cause hospitalization rates per-patient-per-month (PPPM) were assessed during the study period and compared between the weighted cohorts using incidence rate ratios (IRRs) derived from a Poisson regression model. Nonparametric bootstrap procedures with 500 replications were used to evaluate statistical significance and 95% confidence intervals (CIs). The median time to first rehospitalization was also assessed.

## Results

### Study sample

**Fig. 2:**
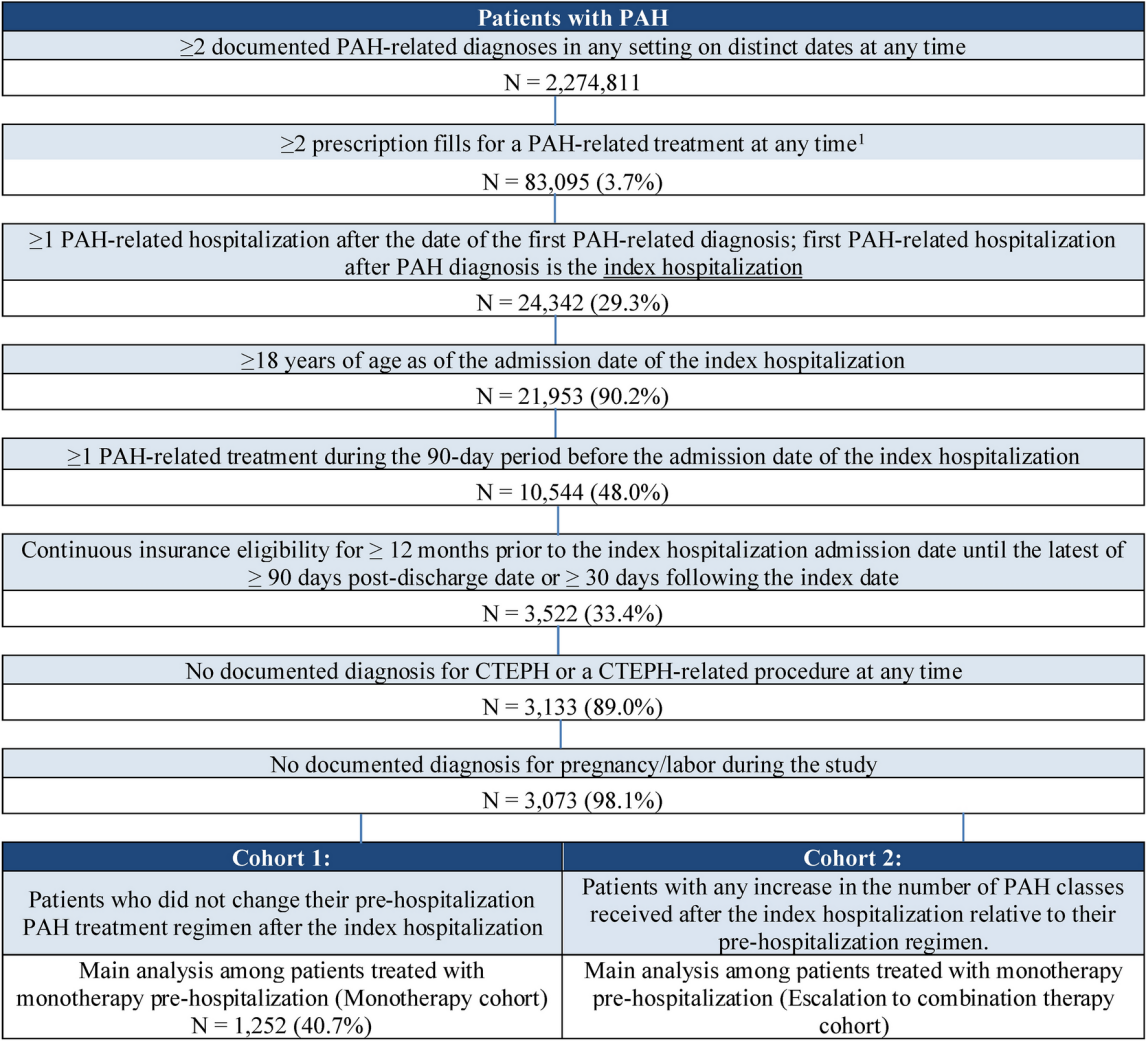
Sample selection. *ERA* Endothelin receptor antagonist, *CTEPH* Chronic thromboembolic pulmonary hypertension, *ICD-10-CM* International Classification of Diseases, 10th Revision, Clinical Modification, *PAH* Pulmonary arterial hypertension, *PDE5i* Phosphodiesterase type 5 inhibitor, *PPA* Prostacyclin pathway agent, *sGCS* Soluble guanylate cyclase stimulator. Notes: [1] PAH-related treatments include PDE5i (sildenafil [excluding dosage corresponding to Viagra] or tadalafil [excluding dosage corresponding to Cialis]), ERA (bosentan, ambrisentan or macitentan), sGCS (riociguat), and PPA (epoprostenol, iloprost, treprostinil or selexipag). [2] Patients with other patterns (i.e., treatment de-escalation or treatment switch) were excluded from the sample. [3] Combination therapy was defined as ≥ 1 day of overlapping days of supply during either the pre- or the post-hospitalization period. The maximum number of treatment classes based on days of supply on a given day in the 90-day pre- or post-hospitalization period was used to define the type of treatment regimen for that period. In instances where a patient had more than one day with the same number of treatment classes within the pre- or post-hospitalization period, the latest of these days in a given period was used to define the treatment regimen and identify study cohorts.

After applying the eligibility criteria, a total of 203 and 1252 patients treated with monotherapy pre-index were included in the Escalation to combination therapy cohort and Monotherapy cohort, respectively (Fig. 2). For the sensitivity analysis among all treated patients pre-index, there were 315 and 2023 patients in each cohort, respectively.

### Baseline characteristics before and after weighting

For monotherapy-treated patients included in the main analysis, baseline characteristics before and after weighting are presented in Table 1 and Supplementary Table S1. Before weighting, patients in the Escalation to combination therapy cohort were on average younger than those in the Monotherapy cohort (mean age: 54.4 vs. 61.9 years) and had generally fewer comorbidities, such as diabetes mellitus (33.0% vs. 44.9%), chronic obstructive pulmonary disease (42.9% vs. 55.4%), and anemia (32.5% vs. 48.7%; all STD > 0.20). Importantly, the Escalation to combination therapy cohort had a higher risk score than the Monotherapy cohort (mean: 11.3 vs. 8.9; STD > 0.20), suggesting a higher risk of unsatisfactory response to treatment in the Escalation to combination therapy cohort. The Escalation to combination therapy cohort also had a higher number of certain PAH-related diagnostic tests including RHC (44.8% vs. 32.3%) and computed tomography angiography (31.0% vs. 19.0%; all STD > 0.20). After weighting, cohorts were well balanced with respect to baseline and index hospitalization characteristics (Table 1, Supplementary Table S1), with all STD < 0.20 with the exception of West and Midwest region (STD: 0.22 and 0.25, respectively). In the sensitivity analysis among all treated patients pre-index, a similar pattern of cohort differences in baseline characteristics was observed before weighting; likewise, all characteristics were well balanced after weighting (all STD < 0.20; Supplementary Table S2).

**Table 1:** Patient characteristics.

Patient characteristics	Monotherapy cohort		Escalation to combination therapy cohort					
			Before weighting		STD	After weighting		STD
	N = 1252		N = 203			N = 203		
Duration of study period, months, mean ± SD	10.0 ± 3.3		9.7 ± 3.4		0.08	9.5 ± 3.5		0.14
At index date								
Age, years, mean ± SD	61.9 ± 14.9		54.4 ± 13.9		0.53^†^	60.8 ± 11.9		0.09
Female, n (%)	853	(68.1)	143	(70.4)	0.05	136	(66.9)	0.03
Region, n (%)								
South	340	(27.2)	73	(36.0)	0.19	57	(28.2)	0.02
West	234	(18.7)	55	(27.1)	0.20^†^	57	(27.9)	0.22^†^
Midwest	285	(22.8)	34	(16.7)	0.15	27	(13.3)	0.25^†^
Northeast	322	(25.7)	31	(15.3)	0.26^†^	51	(25.3)	0.01
Unknown	71	(5.7)	10	(4.9)	0.03	11	(5.2)	0.02
White, n (%)	609	(48.6)	99	(48.8)	0.00	105	(51.7)	0.06
Unknown race	306	(24.4)	57	(28.1)	0.08	55	(26.9)	0.06
Insurance type, n (%)								
Commercial	333	(26.6)	67	(33.0)	0.14	69	(33.9)	0.16
Medicaid	305	(24.4)	65	(32.0)	0.17	50	(24.7)	0.01
Medicare	403	(32.2)	32	(15.8)	0.39^†^	54	(26.7)	0.12
Other	126	(10.1)	31	(15.3)	0.16	24	(11.8)	0.06
Unknown	85	(6.8)	8	(3.9)	0.13	6	(2.9)	0.18
During the baseline period								
Quan-Charlson comorbidity index, mean ± SD	4.1 ± 2.3		3.7 ± 2.1		0.19	4.1 ± 2.3		0.01
Simplified PAH risk score, mean ± SD	8.9 ± 7.9		11.3 ± 8.6		0.30^†^	9.6 ± 8.4		0.08
Comorbidities, n (%)								
Cardiopulmonary comorbidities	1160	(92.7)	178	(87.7)	0.17	185	(90.9)	0.06
Systemic hypertension	1028	(82.1)	152	(74.9)	0.18	168	(82.6)	0.01
Diabetes mellitus	562	(44.9)	67	(33.0)	0.25^†^	82	(40.3)	0.09
Coronary artery disease	555	(44.3)	72	(35.5)	0.18	81	(39.7)	0.09
Obesity	526	(42.0)	75	(36.9)	0.10	78	(38.6)	0.07
Interstitial lung disease	310	(24.8)	45	(22.2)	0.06	41	(20.1)	0.11
Congestive heart failure	912	(72.8)	141	(69.5)	0.07	160	(78.9)	0.14
Chronic obstructive pulmonary disease	693	(55.4)	87	(42.9)	0.25^†^	114	(56.1)	0.01
Anemia	610	(48.7)	66	(32.5)	0.33^†^	91	(44.7)	0.08
Hypothyroidism	329	(26.3)	43	(21.2)	0.12	56	(27.8)	0.03
Connective tissue disease/rheumatic disease	273	(21.8)	57	(28.1)	0.15	44	(21.6)	0.00
RHC procedures, n (%)	403	(32.2)	91	(44.8)	0.26^†^	69	(33.9)	0.04
Number of treatment classes, n (%)	1.1 ± 0.3		1.2 ± 0.4		0.37^†^	1.1 ± 0.3		0.04
Selected pharmacological treatments, n (%)								
Antihypertensives	1123	(89.7)	187	(92.1)	0.08	183	(90.4)	0.02
Anticoagulants	472	(37.7)	33	(16.3)	0.50^†^	70	(34.7)	0.06
≥ 1 all-cause inpatient stay, n (%)	784	(62.6)	110	(54.2)	0.17	125	(61.8)	0.02
Time from first PAH-related treatment to the index hospitalization admission date (days), mean ± SD	602.7 ± 507.8		500.3 ± 545.8		0.19	600.3 ± 555.5		0.00
Index hospitalization LOS (days), mean ± SD	9.2 ± 14.4		10.4 ± 13.9		0.09	10.6 ± 12.3		0.10
Time between discharge date and index date (days), mean ± SD	30.3 ± 25.3		35.0 ± 25.8		0.18	32.4 ± 24.6		0.08

^†^STD > 0.2

*LOS* length of stay, *PAH* pulmonary arterial hypertension, *RHC* right heart catheterization, *SD* standard deviation, *STD* standardized difference.

### PAH treatment patterns

**Fig. 3:**
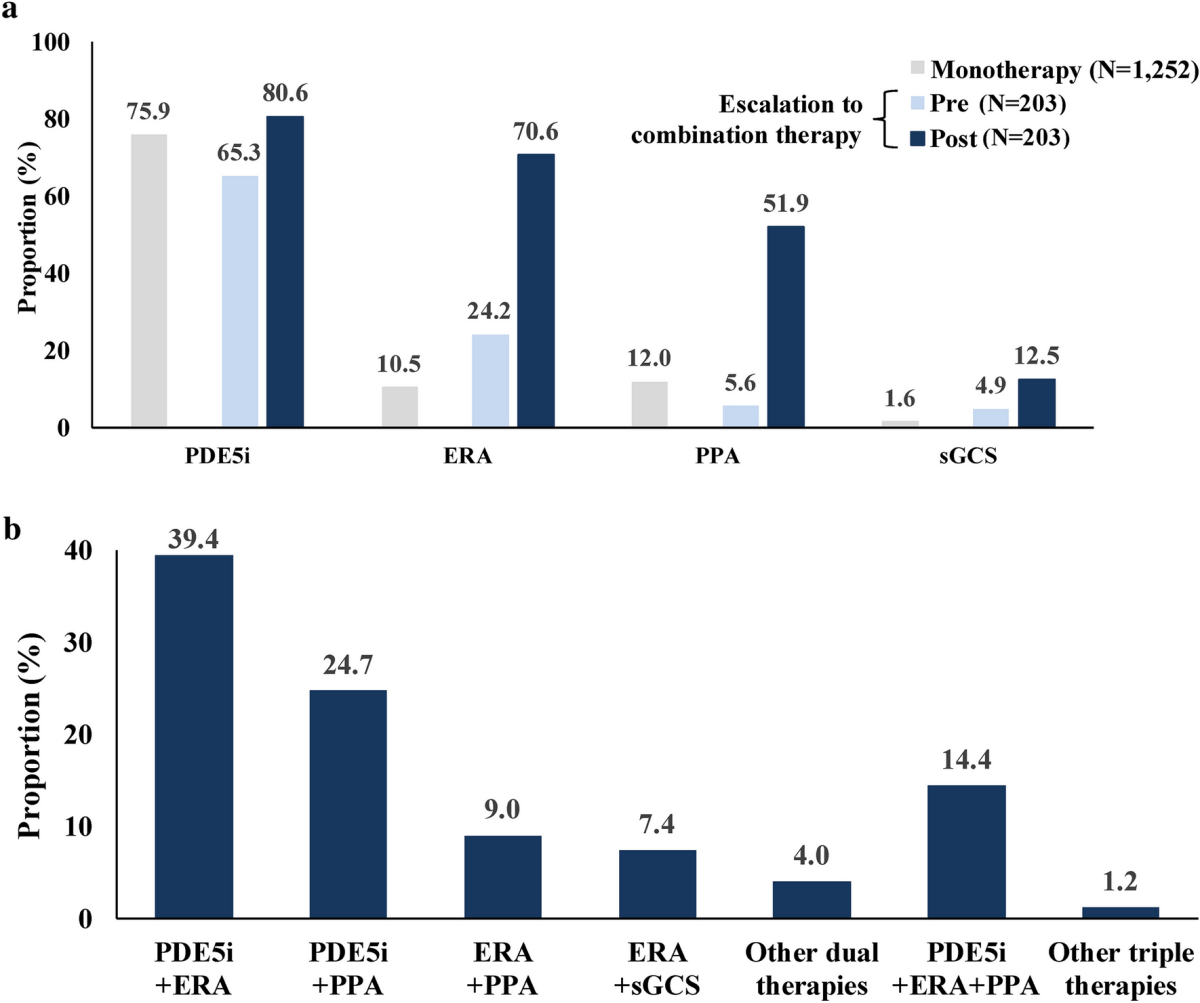
Treatment patterns in the pre- and post-hospitalization period among weighted cohorts. (**a**) Proportion of patients receiving a given treatment class during the pre- and post-hospitalization period among the weighted Monotherapy and Escalation to combination therapy cohorts^1^. (**b**) Proportion of patients in the Escalation to combination therapy cohort receiving a given combination therapy during the post-hospitalization period. *ERA* Endothelin receptor antagonist, *PDE5i* Phosphodiesterase type 5 inhibitor, *PPA* Prostacyclin pathway agent, *sGCS* Soluble guanylate cyclase stimulator. Note: [1] Treatment classes are not mutually exclusive, as patients may have received more than one treatment class. Thus, for the Monotherapy and Escalation to combination therapy cohorts, the proportion of patients across treatment classes do not necessarily sum to 100%.

Prior to the index date, all patients were treated with monotherapy, with PDE5i being the most common treatment for both cohorts after weighting (Escalation to combination therapy cohort: 65.3%; Monotherapy cohort: 75.9%; Fig. 3). Although there was an increase in the prescription for all drug classes after hospitalization, this was more pronounced for ERAs (24.2% before vs. 70.6% after hospitalization) and PPAs (5.6% before vs. 51.9% after hospitalization; Fig. 3a). During the post-hospitalization period, 84.5% of the Escalation to combination therapy cohort augmented to double oral therapy and 15.5% augmented to triple therapy or more, with the most common combination therapies being PDE5i + ERA (39.4%), PDE5i + PPA (24.7%), and PDE5i + ERA + PPA (14.4%; Fig. 3b). A similar pattern of results was obtained in the sensitivity analysis (Supplementary Table S3).

### All-cause rehospitalization

**Fig. 4:**
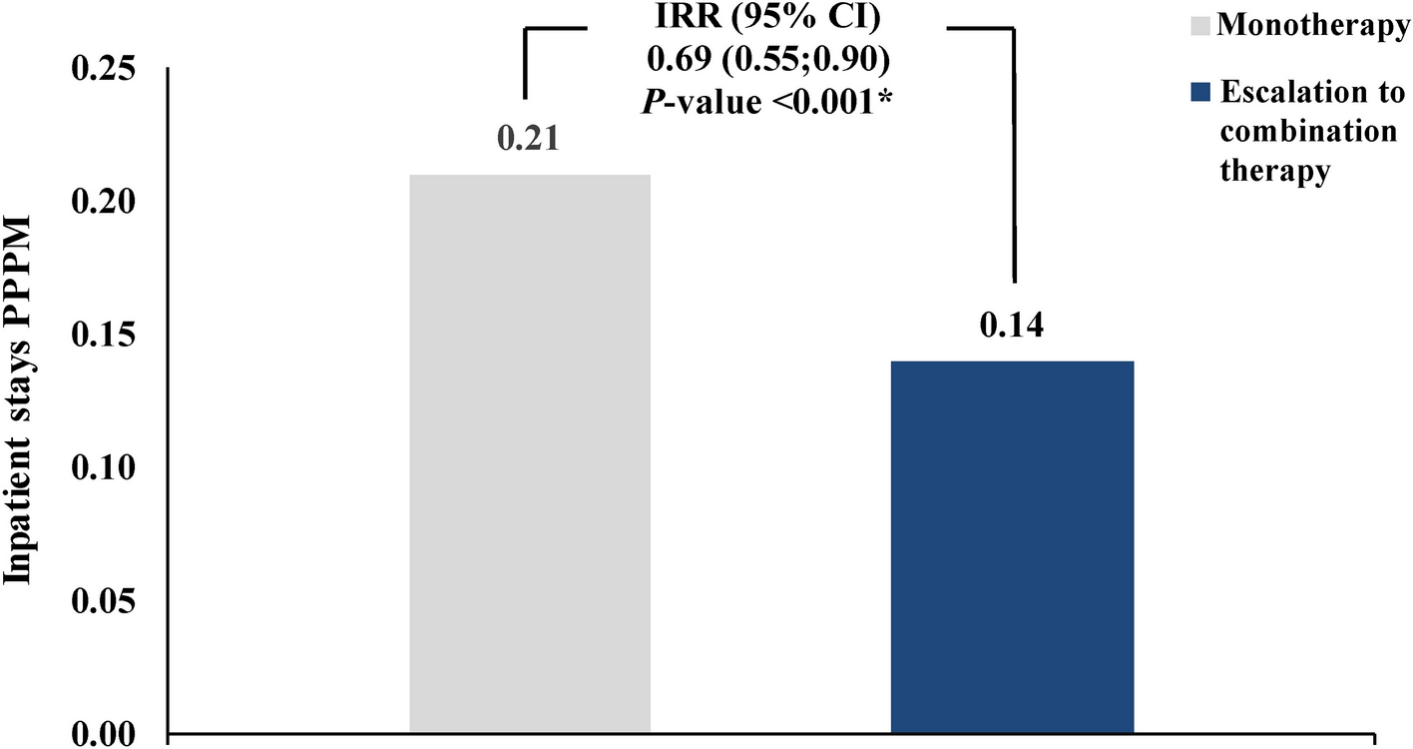
Hospitalization rates among weighted cohorts. *CI* Confidence interval, *IRR* Incidence rate ratio, *PPPM* Per-patient-per-month.

In weighted cohorts, the rate of all-cause rehospitalization was 31% lower in the Escalation to combination therapy cohort compared to the Monotherapy cohort (mean rate PPPM: 0.14 vs. 0.21; IRR [95% CI]: 0.69 [0.55, 0.90]; p < 0.001; Fig. 4). The most common primary diagnoses for rehospitalization in the Escalation to combination therapy cohort and Monotherapy cohort were related to cardiopulmonary events, with shortness of breath (11.7% and 16.0%, respectively), unspecified pulmonary hypertension (16.8% and 11.7%), and unspecified heart failure (12.0% and 5.5%; Supplementary Table S4) being the most common for both cohorts. The median time to first rehospitalization was numerically longer for the Escalation to combination therapy cohort than for the Monotherapy cohort (217 vs. 176 days). In the sensitivity analysis among all treated patients, a similar reduction in rehospitalization rates was observed among the Escalation cohort compared to the No treatment change cohort (mean rate PPPM: 0.15 vs. 0.18; IRR [95% CI]: 0.81 [0.66, 0.99]; p = 0.040; Supplementary Fig. S1). Similarly, the most common primary diagnoses for rehospitalization were related to cardiopulmonary events (Supplementary Table S5). The median time to first rehospitalization was also numerically longer for the Escalation cohort than for the No treatment change cohort (224 vs. 209 days).

## Discussion

The present study assessed the impact of treatment escalation on rehospitalization rates among treated PAH patients who had been hospitalized for PAH-related reasons in the US, with a particular focus among patients treated with monotherapy. Prior to their first hospitalization, most patients were treated with PDE5i monotherapy. Among patients who underwent treatment escalation post-hospitalization, the most common combination therapies were PDE5i + ERA, PDE5i + PPA, and PDE5i + ERA + PPA. Rates of rehospitalization were lower among patients who escalated their treatment compared to those who remained on the same treatment, for both groups of monotherapy-treated patients and any treated patients pre-index (IRR: 0.69 and 0.81, respectively, all p < 0.05). Thus, our findings indicate that treatment escalation may curb the frequency of rehospitalization among PAH patients, with pronounced results for patients initially treated with monotherapy escalating to combination therapy.

Our findings are highly relevant given the persistent gap between PAH clinical practice and current ESC/ERS treatment guidelines^4,7,14,33^, which recommend combination therapy for many patients (e.g., patients without cardiopulmonary comorbidities) along with regular follow-up assessment and the addition of a PPA should that patient not achieve low risk status^21^. In contrast, our findings and others show the predominance of monotherapy over combination therapy in real-world settings^7,14,33^. In our study, only 1 in 10 treated patients (i.e., 315/3073) escalated their treatment post-hospitalization, consistent with prior data suggesting that hospitalization does not prompt any change in PAH treatment strategy^14^. For instance, Ogbomo et al.^14^ reported that at least half of PAH patients in the US were prescribed monotherapy during both the pre- and post-hospitalization periods (64.8% vs. 51.9%), between 2014 and 2019. Furthermore, the majority of patients had no therapy modification post-hospitalization (72.8%), while only a minority escalated therapy (6.1%)^14^. This predominant reliance on monotherapy suggests a stagnation in PAH therapy management over time. A similar pattern has been observed during an earlier study period (2004–2006), in which only 12.7% of all patients augmented their therapy over a mean follow-up of 1.4 years after their first claim for a PAH medication^7^. Evidently, a paradigm shift in PAH clinical practice is warranted based on contemporary guidelines and the latest available data, which recommend the use of initial combination therapy for low-to-intermediate risk treatment-naïve patients without cardiopulmonary comorbidities and treatment escalation among those who are high-risk^21^. A caveat is that real-world patients are more likely to have comorbidities than those in clinical trials^46^, which may disqualify them from combination therapy per ESC/ERS guidelines^21^. Thus, the high rate of monotherapy use in the present study might reflect the fact that not all patients were suitable candidates for combination therapy. That said, evidence from the AMBITION and GRIPHON trials suggest that combination therapy may improve clinical outcomes among patients with cardiopulmonary comorbidities relative to monotherapy and placebo, respectively^30,47^, although the response to initial combination therapy was weaker among patients with multiple risk factors for left ventricular diastolic dysfunction^48^. Furthermore, the ESC/ERS guidelines have certain limitations, as there is still no clear consensus on how to define PAH patients with comorbidities, nor is there any clear distinction based on the severity and type of comorbidity or whether the comorbidity is controlled^21,49^. Nonetheless, the high rate of monotherapy use post-hospitalization suggests that hospitalization remains a missed opportunity to adjust therapy, albeit a late one, considering the strong evidence from clinical trials showing that proactive use of combination therapy can prevent disease progression events including hospitalization^23,24,37^.

Prior to reweighting the cohorts through entropy balancing, the patients in our study who remained on monotherapy after hospitalization were of older age and had more comorbidities, but had lower simplified claims-based risk score^44^. This may reflect potential tolerability issues among older patients or patients with multiple comorbidities^50–52^. Conversely, more treatment escalation to combination therapy among patients with high-risk disease is consistent with current guideline recommendations. However, it should be noted that the simplified claims-based risk score is only a proxy for unsatisfactory response to treatment based on information available in claims and may not fully capture the true PAH severity. Therefore, it is possible that patients who escalated to combination therapy presented with a more severe risk profile at the outset of the study despite adjustment for confounding variables. If these higher-risk patients were also more likely to have hospitalizations, this could have led to an underestimation of the association between treatment escalation and reduced rehospitalization rates.

Our results suggest that a shift away from monotherapy towards a greater reliance on combination therapy might help to reduce the risk of rehospitalization in PAH. These findings are in turn corroborated by evidence from prior clinical and real-world studies. For instance, meta-analyses have found that combination therapies significantly reduce the risk for the composite endpoint of clinical worsening when compared to monotherapy, and this effect was largely driven by a reduction in non-fatal events such as hospitalization^25,26^. Aside from the impact on clinical worsening as a composite endpoint, one meta-analysis also found that combination therapy reduced the risk of PAH-related hospital admission by 29%^26^. To date, there is limited real-world evidence regarding the impact of PAH regimen on readmission rates following a PAH-related hospitalization. However, analyses of data from the US PharMetrics Database (2011–2015) have found that PAH regimens containing a PPA reduced/delayed hospital readmissions compared to other regimens, and this was the case for patients continuing or initiating a new PPA^53^. Another study using the PharMetrics database (2011–2015) found that higher adherence to PAH medications was associated with a lower risk of rehospitalization, and the reduction in risk was more pronounced for ERAs compared to PDE5i^54^. Our study builds on this prior evidence by showing that treatment escalation to combination therapy generally reduces the risk of rehospitalization relative to continuation of monotherapy. However, our findings may also speak to the benefits of ERA and PPA, as these were the most commonly added classes among patients who escalated to combination therapy. Furthermore, reduced rehospitalization may also be attributed to improvements in hemodynamics and/or clinical risk parameters^55,56^, although risk scores have been previously shown to be surrogates of long-term clinical outcomes and not endpoints in and of themselves^57^. Whether the reduction in rehospitalization rates observed in our study is attributable directly to treatment escalation to combination therapy or through other mechanisms remains to be elucidated. Further investigation, for instance, by comparing hemodynamic parameters and/or clinical risk scores at final follow-up between patients with treatment escalation to combination therapy and those who continued monotherapy, is warranted.

The present findings have important implications from a clinical standpoint. In particular, escalating PAH therapy post-hospitalization may be especially critical given that hospitalization is associated with progression of PAH, early mortality, and hospital readmission^3–13,34^. In a REVEAL registry study of > 800 patients with diagnosed PAH evaluated for first-time PAH-related hospitalization^6^, only one third of patients remained hospitalization-free for 3 years and nearly half did not survive 3 years post-discharge^6^. These findings have been corroborated by more recent studies reporting high rates of rehospitalization following a PAH-related hospitalization; additionally, prior studies suggest that greater PAH severity predicts worse outcomes^34,36,58^. For instance, studies have found that approximately one quarter of patients with PAH were rehospitalized within 30 days of a PAH-related hospitalization^34,36,58^. Additionally, higher CCI and the presence of specific comorbidities (e.g., obesity, congestive heart failure, diabetes, chronic lung disease, etc.) have been associated with an increased risk of 30-day readmission^34^. Consistent with our findings, prior studies have also found that cardiopulmonary events are common causes for hospital readmission^34,59^. Overall, these studies show that rehospitalization is frequent and strategies aiming at reducing it, such as early treatment escalation to combination therapy, could be an option to mitigate the disease burden.

From an economic perspective, the burden of PAH is substantial in terms of HRU and costs in the US^3–13,60^. In 2022, average monthly expenses were estimated at $15,686 per patient with PAH^11^. Frequent hospitalizations have been identified as a primary driver of this high economic burden^3–15^. Furthermore, analyses of temporal changes in PAH care suggest that the costs associated with hospitalization and the length of hospital stay have been increasing substantially over time^8,10^. Thus, treatment escalation to combination therapy may help to alleviate the downstream burden on the healthcare system and contain costs from a healthcare payer perspective. While combination therapy for PAH is generally more costly than monotherapy, these higher pharmacy costs may be at least partly offset by reduced medical costs^3,4,61,62^. Consistent with this, prior evidence has shown that average total healthcare costs among patients with PAH were largely driven by hospitalization costs (between 40 and 60% of total costs across studies), and to a lesser extent by pharmacy costs (between 15 and 40% of total costs across studies)^5,7,11–13,63^. Thus, given the current availability of highly effective treatments that can reduce the burden of hospitalization, it is critical to raise clinicians’ awareness and improve patient access to the full spectrum of PAH therapeutics. Although treatment escalation to combination therapy may reduce rehospitalizations among all patients with PAH, this strategy may be particularly beneficial for those initiated on monotherapy and/or those with more severe disease who are at greater risk of hospitalization from the outset.

### Limitations

The present study was subject to certain limitations. First, patients were classified into study cohorts based on information available in health insurance claims data such as diagnosis codes and treatment received. Consequently, patients may have been misclassified in a given cohort in cases where information was recorded incorrectly. For instance, it is possible that some patients might have been incorrectly diagnosed with PAH and treated with a PAH-related medication when in fact they might have had other types of pulmonary hypertension, such as Groups 2–5 instead of Group 1. Second, although comparative analyses were adjusted for observable patient characteristics, one cannot completely rule out the possibility of unmeasured confounding due to unobservable confounders. Further, certain observable characteristics remained slightly imbalanced after reweighting (e.g., region), which may have confounded the results. Third, hemodynamic and clinical risk parameters are not available in claims data; thus, the mechanism through which combination therapy had an impact on rehospitalization could not be assessed. Finally, the study results may not be generalizable to patients without health insurance, as well as to patients with PAH who were not hospitalized due to PAH or who were not treated for PAH.

## Conclusions

Although highly effective combination therapies are currently available and recommended for the treatment of PAH, the use of monotherapy remains pervasive in real-world settings. This gap between PAH clinical practice and current guidelines recommendations is concerning given the substantial burden of disease in PAH. In light of these ongoing challenges, the present study is one of the first to assess the impact of treatment escalation on rehospitalization rates among patients following a PAH-related hospitalization. Our results show that patients with treatment escalation post-hospitalization had a lower rehospitalization rate when compared to those who remained on the same treatment, particularly if that treatment was monotherapy. Given that hospitalizations are a key driver of economic burden and a marker of disease progression in PAH, earlier treatment escalation and the broader use of combination therapy to target multiple disease pathways has the potential to alleviate the substantial burden associated with PAH.

## Supplementary Information

Supplementary Information.

## Data Availability

The data that support the findings of this study are available from KRD. Restrictions apply to the availability of these data, which were used under license for this study. Data are available from the authors upon reasonable request and with the permission of KRD.
