# Supplementary material for: Impact of treatment escalation on rehospitalization among patients with pulmonary arterial hypertension
Source: Sci Rep. 2025 Apr 10;15:12235. doi: 10.1038/s41598-025-90975-4 (PMC11986031; doi:10.1038/s41598-025-90975-4)
Supplement: Supplementary file 1 — Supplementary Information. [file 41598_2025_90975_MOESM1_ESM.docx]

# Supplementary materials

## Table S1. Patient characteristics (full list)

|  | **Monotherapy cohort** | | **Escalation to combination therapy cohort** | | | | | | | |
| --- | --- | --- | --- | --- | --- | --- | --- | --- | --- | --- |
| **Patient characteristics** |  |  | **Before weighting** | | **STD** | | **After weighting** | | **STD** | |
|  | **N = 1,252** | | **N = 203** | | | | **N = 203** | | | |
| **Duration of study period, months, mean ± SD** | 10.0 ± 3.3 | | 9.7 ± 3.4 | | 0.08 |  | 9.5 ± 3.5 | | 0.14 |  |
| ***At index date*** |  | |  | |  |  |  | |  |  |
| **Age, years, mean ± SD** | 61.9 ± 14.9 | | 54.4 ± 13.9 | | 0.53 | † | 60.8 ± 11.9 | | 0.09 |  |
| **Female, n (%)** | 853 | (68.1) | 143 | (70.4) | 0.05 |  | 136 | (66.9) | 0.03 |  |
| **Region, n (%)** |  |  |  |  |  |  |  |  |  |  |
| South | 340 | (27.2) | 73 | (36.0) | 0.19 |  | 57 | (28.2) | 0.02 |  |
| West | 234 | (18.7) | 55 | (27.1) | 0.20 | † | 57 | (27.9) | 0.22 | † |
| Midwest | 285 | (22.8) | 34 | (16.7) | 0.15 |  | 27 | (13.3) | 0.25 | † |
| Northeast | 322 | (25.7) | 31 | (15.3) | 0.26 | † | 51 | (25.3) | 0.01 |  |
| Unknown | 71 | (5.7) | 10 | (4.9) | 0.03 |  | 11 | (5.2) | 0.02 |  |
| **Race, n (%)** |  |  |  |  |  |  |  |  |  |  |
| White | 609 | (48.6) | 99 | (48.8) | 0.00 |  | 105 | (51.7) | 0.06 |  |
| Black or African American | 254 | (20.3) | 38 | (18.7) | 0.04 |  | 34 | (16.8) | 0.09 |  |
| American Indian or Alaska Native | 1 | (0.1) | 0 | (0.0) | 0.04 |  | 0 | (0.0) | 0.04 |  |
| Asian or Pacific Islander | 26 | (2.1) | 3 | (1.5) | 0.05 |  | 4 | (2.1) | 0.00 |  |
| Other | 56 | (4.5) | 6 | (3.0) | 0.08 |  | 5 | (2.4) | 0.11 |  |
| Unknown | 306 | (24.4) | 57 | (28.1) | 0.08 |  | 55 | (26.9) | 0.06 |  |
| **Insurance type, n (%)** |  |  |  |  |  |  |  |  |  |  |
| Commercial | 333 | (26.6) | 67 | (33.0) | 0.14 |  | 69 | (33.9) | 0.16 |  |
| Medicaid | 305 | (24.4) | 65 | (32.0) | 0.17 |  | 50 | (24.7) | 0.01 |  |
| Medicare | 403 | (32.2) | 32 | (15.8) | 0.39 | † | 54 | (26.7) | 0.12 |  |
| Other | 126 | (10.1) | 31 | (15.3) | 0.16 |  | 24 | (11.8) | 0.06 |  |
| Unknown | 85 | (6.8) | 8 | (3.9) | 0.13 |  | 6 | (2.9) | 0.18 |  |
| **Year of index hospitalization admission, n (%)** |  |  |  |  |  |  |  |  |  |  |
| 2016 | 40 | (3.2) | 6 | (3.0) | 0.01 |  | 7 | (3.2) | 0.00 |  |
| 2017 | 223 | (17.8) | 29 | (14.3) | 0.10 |  | 38 | (18.7) | 0.02 |  |
| 2018 | 334 | (26.7) | 54 | (26.6) | 0.00 |  | 42 | (20.5) | 0.15 |  |
| 2019 | 242 | (19.3) | 40 | (19.7) | 0.01 |  | 44 | (21.7) | 0.06 |  |
| 2020 | 198 | (15.8) | 30 | (14.8) | 0.03 |  | 30 | (15.0) | 0.02 |  |
| 2021 | 215 | (17.2) | 44 | (21.7) | 0.11 |  | 42 | (20.8) | 0.09 |  |
| ***During the baseline period*** |  | |  | |  |  |  | |  |  |
| **Quan-Charlson comorbidity index, mean ± SD** | 4.1 ± 2.3 | | 3.7 ± 2.1 | | 0.19 |  | 4.1 ± 2.3 | | 0.01 |  |
| **Simplified PAH risk score, mean ± SD** | 8.9 ± 7.9 | | 11.3 ± 8.6 | | 0.30 | † | 9.6 ± 8.4 | | 0.08 |  |
| **Comorbidities, n (%)** |  |  |  |  |  |  |  |  |  |  |
| Cardiopulmonary comorbidities | 1,160 | (92.7) | 178 | (87.7) | 0.17 |  | 185 | (90.9) | 0.06 |  |
| Systemic hypertension | 1,028 | (82.1) | 152 | (74.9) | 0.18 |  | 168 | (82.6) | 0.01 |  |
| Diabetes mellitus | 562 | (44.9) | 67 | (33.0) | 0.25 | † | 82 | (40.3) | 0.09 |  |
| Coronary artery disease | 555 | (44.3) | 72 | (35.5) | 0.18 |  | 81 | (39.7) | 0.09 |  |
| Obesity | 526 | (42.0) | 75 | (36.9) | 0.10 |  | 78 | (38.6) | 0.07 |  |
| Interstitial lung disease | 310 | (24.8) | 45 | (22.2) | 0.06 |  | 41 | (20.1) | 0.11 |  |
| Congestive heart failure | 912 | (72.8) | 141 | (69.5) | 0.07 |  | 160 | (78.9) | 0.14 |  |
| Chronic obstructive pulmonary disease | 693 | (55.4) | 87 | (42.9) | 0.25 | † | 114 | (56.1) | 0.01 |  |
| Anemia | 610 | (48.7) | 66 | (32.5) | 0.33 | † | 91 | (44.7) | 0.08 |  |
| Renal disease | 520 | (41.5) | 54 | (26.6) | 0.32 | † | 70 | (34.3) | 0.15 |  |
| Depression | 379 | (30.3) | 52 | (25.6) | 0.10 |  | 54 | (26.7) | 0.08 |  |
| Hypothyroidism | 329 | (26.3) | 43 | (21.2) | 0.12 |  | 56 | (27.8) | 0.03 |  |
| Asthma | 305 | (24.4) | 43 | (21.2) | 0.08 |  | 43 | (21.3) | 0.07 |  |
| Connective tissue disease/rheumatic disease | 273 | (21.8) | 57 | (28.1) | 0.15 |  | 44 | (21.6) | 0.00 |  |
| **PAH-related procedures, n (%)** |  |  |  |  |  |  |  |  |  |  |
| ECG (chest) | 1,056 | (84.3) | 164 | (80.8) | 0.09 |  | 173 | (85.2) | 0.02 |  |
| Echocardiography (chest) | 1,007 | (80.4) | 172 | (84.7) | 0.11 |  | 163 | (80.4) | 0.00 |  |
| Computed tomography (chest) | 490 | (39.1) | 84 | (41.4) | 0.05 |  | 83 | (40.8) | 0.03 |  |
| RHC | 403 | (32.2) | 91 | (44.8) | 0.26 | † | 69 | (33.9) | 0.04 |  |
| Computed tomography angiography | 238 | (19.0) | 63 | (31.0) | 0.28 | † | 51 | (25.2) | 0.15 |  |
| **Number of treatment classes, n (%)** | 1.1 ± 0.3 | | 1.2 ± 0.4 | | 0.37 | † | 1.1 ± 0.3 | | 0.04 |  |
| **Selected pharmacological treatments, n (%)** |  |  |  |  |  |  |  |  |  |  |
| Antidepressants | 458 | (36.6) | 64 | (31.5) | 0.11 |  | 69 | (33.8) | 0.06 |  |
| Antihypertensives | 1,123 | (89.7) | 187 | (92.1) | 0.08 |  | 183 | (90.4) | 0.02 |  |
| Anticoagulants | 472 | (37.7) | 33 | (16.3) | 0.50 | † | 70 | (34.7) | 0.06 |  |
| **≥1 all-cause inpatient stay, n (%)** | 784 | (62.6) | 110 | (54.2) | 0.17 |  | 125 | (61.8) | 0.02 |  |
| **Time from first observed PAH-related diagnosis to the index hospitalization admission date (days), mean ± SD** | 672.9 ± 485.8 | | 587.4 ± 519.3 | | 0.17 |  | 656.7 ± 519.2 | | 0.03 |  |
| **Time from first PAH-related treatment to the index hospitalization admission date (days), mean ± SD** | 602.7 ± 507.8 | | 500.3 ± 545.8 | | 0.19 |  | 600.3 ± 555.5 | | 0.00 |  |
| **Index hospitalization LOS (days), mean ± SD** | 9.2 ± 14.4 | | 10.4 ± 13.9 | | 0.09 |  | 10.6 ± 12.3 | | 0.10 |  |
| **Time between discharge date and index date (days), mean ± SD** | 30.3 ± 25.3 | | 35.0 ± 25.8 | | 0.18 |  | 32.4 ± 24.6 | | 0.08 |  |

† standardized difference >0.2

**Abbreviations:** LOS: length of stay; PAH: pulmonary arterial hypertension; RHC: right heart catheterization; SD: standard deviation; STD: standardized difference.

## Table S2. Patient characteristics (sensitivity analysis among any treated patients during the pre-index hospitalization period)

| **Patient characteristics** | **No treatment change cohort** | | **Escalation cohort** | | | | | | | | | |  |
| --- | --- | --- | --- | --- | --- | --- | --- | --- | --- | --- | --- | --- | --- |
|  |  |  | **Before weighting** | | **STD** | | | | **After weighting** | | | **STD** |  |
|  | **N = 2,023** | | **N = 315** | | | | | | **N = 315** | | | |  |
| **Duration of study period, months, mean ± SD** | 10.1 ± 3.2 | | 9.8 ± 3.3 | | 0.07 | |  | | 9.8 ± 3.3 | | | 0.08 |  |
| ***At index date*** |  | |  | |  | |  | |  | | |  |  |
| **Age, years, mean ± SD** | 59.5 ± 14.8 | | 53.5 ± 13.7 | | 0.42 | | † | | 58.4 ± 12.3 | | | 0.08 |  |
| **Female, n (%)** | 1,444 | (71.4) | 237 | (75.2) | | 0.09 | |  | | 223 | (70.7) | 0.02 |  |
| **Region, n (%)** |  |  |  |  | |  | |  | |  |  |  |  |
| South | 596 | (29.5) | 111 | (35.2) | | 0.12 | |  | | 104 | (33.0) | 0.08 |  |
| West | 415 | (20.5) | 86 | (27.3) | | 0.16 | |  | | 84 | (26.5) | 0.14 |  |
| Midwest | 432 | (21.4) | 63 | (20.0) | | 0.03 | |  | | 50 | (15.8) | 0.14 |  |
| Northeast | 480 | (23.7) | 43 | (13.7) | | 0.26 | | † | | 67 | (21.4) | 0.06 |  |
| Unknown | 100 | (4.9) | 12 | (3.8) | | 0.06 | |  | | 10 | (3.2) | 0.09 |  |
| **Race, n (%)** |  |  |  |  | |  | |  | |  |  |  |  |
| White | 983 | (48.6) | 160 | (50.8) | | 0.04 | |  | | 159 | (50.5) | 0.04 |  |
| Black or African American | 373 | (18.4) | 51 | (16.2) | | 0.06 | |  | | 51 | (16.2) | 0.06 |  |
| American Indian or Alaska Native | 3 | (0.1) | 0 | (0.0) | | 0.05 | |  | | 0 | (0.0) | 0.05 |  |
| Asian or Pacific Islander | 47 | (2.3) | 6 | (1.9) | | 0.03 | |  | | 6 | (2.0) | 0.02 |  |
| Other | 78 | (3.9) | 7 | (2.2) | | 0.10 | |  | | 8 | (2.6) | 0.07 |  |
| Unknown | 539 | (26.6) | 91 | (28.9) | | 0.05 | |  | | 90 | (28.7) | 0.05 |  |
| **Insurance type, n (%)** |  |  |  |  | |  | |  | |  |  |  |  |
| Commercial | 567 | (28.0) | 98 | (31.1) | | 0.07 | |  | | 99 | (31.5) | 0.08 |  |
| Medicaid | 530 | (26.2) | 112 | (35.6) | | 0.20 | | † | | 95 | (30.1) | 0.09 |  |
| Medicare | 551 | (27.2) | 45 | (14.3) | | 0.32 | | † | | 69 | (21.8) | 0.13 |  |
| Other | 258 | (12.8) | 50 | (15.9) | | 0.09 | |  | | 42 | (13.5) | 0.02 |  |
| Unknown | 117 | (5.8) | 10 | (3.2) | | 0.13 | |  | | 10 | (3.1) | 0.13 |  |
| **Year of index hospitalization admission date, n (%)** |  |  |  |  | |  | |  | |  |  |  |  |
| 2016 | 73 | (3.6) | 11 | (3.5) | | 0.01 | |  | | 13 | (4.0) | 0.02 |  |
| 2017 | 382 | (18.9) | 47 | (14.9) | | 0.11 | |  | | 54 | (17.1) | 0.05 |  |
| 2018 | 547 | (27.0) | 85 | (27.0) | | 0.00 | |  | | 83 | (26.4) | 0.01 |  |
| 2019 | 391 | (19.3) | 58 | (18.4) | | 0.02 | |  | | 62 | (19.6) | 0.01 |  |
| 2020 | 302 | (14.9) | 49 | (15.6) | | 0.02 | |  | | 38 | (12.1) | 0.08 |  |
| 2021 | 328 | (16.2) | 65 | (20.6) | | 0.11 | |  | | 66 | (20.9) | 0.12 |  |
| ***During the baseline period*** |  | |  | |  | |  | |  | | |  |  |
| **Quan-Charlson comorbidity index, mean ± SD** | 3.9 ± 2.4 | | 3.6 ± 2.2 | | 0.15 | |  | | 3.9 ± 2.4 | | | 0.02 |  |
| **Simplified PAH risk score, mean ± SD** | 8.6 ± 7.4 | | 10.5 ± 8.1 | | 0.25 | | † | | 8.6 ± 8.1 | | | 0.01 |  |
| **Comorbidities, n (%)** |  |  |  |  | |  | |  | |  |  |  |  |
| Cardiopulmonary comorbidities | 1,788 | (88.4) | 259 | (82.2) | | 0.17 | |  | | 271 | (86.1) | 0.07 |  |
| Systemic hypertension | 1,536 | (75.9) | 218 | (69.2) | | 0.15 | |  | | 239 | (75.7) | 0.00 |  |
| Diabetes mellitus | 785 | (38.8) | 89 | (28.3) | | 0.22 | | † | | 109 | (34.6) | 0.09 |  |
| Coronary artery disease | 735 | (36.3) | 91 | (28.9) | | 0.16 | |  | | 105 | (33.4) | 0.06 |  |
| Obesity | 782 | (38.7) | 106 | (33.7) | | 0.10 | |  | | 110 | (35.0) | 0.08 |  |
| Interstitial lung disease | 470 | (23.2) | 74 | (23.5) | | 0.01 | |  | | 63 | (20.1) | 0.08 |  |
| Congestive heart failure | 1,415 | (69.9) | 218 | (69.2) | | 0.02 | |  | | 231 | (73.2) | 0.07 |  |
| Chronic obstructive pulmonary disease | 1,008 | (49.8) | 125 | (39.7) | | 0.21 | | † | | 129 | (41.0) | 0.18 |  |
| Anemia | 896 | (44.3) | 114 | (36.2) | | 0.17 | |  | | 119 | (37.8) | 0.13 |  |
| Renal disease | 705 | (34.8) | 75 | (23.8) | | 0.24 | | † | | 90 | (28.5) | 0.14 |  |
| Depression | 575 | (28.4) | 86 | (27.3) | | 0.03 | |  | | 88 | (28.1) | 0.01 |  |
| Hypothyroidism | 511 | (25.3) | 70 | (22.2) | | 0.07 | |  | | 68 | (21.5) | 0.09 |  |
| Asthma | 471 | (23.3) | 65 | (20.6) | | 0.06 | |  | | 64 | (20.5) | 0.07 |  |
| Connective tissue disease/rheumatic disease | 469 | (23.2) | 95 | (30.2) | | 0.16 | |  | | 74 | (23.6) | 0.01 |  |
| **PAH-related procedures, n (%)** |  |  |  |  | |  | |  | |  |  |  |  |
| ECG (chest) | 1,625 | (80.3) | 248 | (78.7) | | 0.04 | |  | | 236 | (74.9) | 0.13 |  |
| Echocardiography (chest) | 1,665 | (82.3) | 272 | (86.3) | | 0.11 | |  | | 252 | (80.0) | 0.06 |  |
| Computed tomography (chest) | 694 | (34.3) | 125 | (39.7) | | 0.11 | |  | | 105 | (33.4) | 0.02 |  |
| RHC | 653 | (32.3) | 136 | (43.2) | | 0.23 | | † | | 103 | (32.5) | 0.01 |  |
| Computed tomography angiography | 375 | (18.5) | 90 | (28.6) | | 0.24 | | † | | 72 | (22.7) | 0.10 |  |
| **Number of PAH treatment classes, n (%)** | 1.5 ± 0.7 | | 1.6 ± 0.7 | | 0.05 | |  | | 1.6 ± 0.7 | | | 0.02 |  |
| **Selected pharmacological treatments, n (%)** |  |  |  |  | |  | |  | |  |  |  |  |
| Antidepressants | 736 | (36.4) | 107 | (34.0) | | 0.05 | |  | | 107 | (34.0) | 0.05 |  |
| Antihypertensives | 1,813 | (89.6) | 282 | (89.5) | | 0.00 | |  | | 290 | (92.0) | 0.08 |  |
| Anticoagulants | 698 | (34.5) | 49 | (15.6) | | 0.45 | | † | | 96 | (30.4) | 0.09 |  |
| **≥1 all-cause inpatient stay, n (%)** | 1,100 | (54.4) | 153 | (48.6) | | 0.12 | |  | | 167 | (53.1) | 0.02 |  |
| **Time from first observed PAH-related diagnosis to the index hospitalization admission date (days), mean ± SD** | 733.2 ± 496.8 | | 652.9 ± 541.4 | | 0.15 | |  | | 735.8 ± 537.3 | | | 0.01 |  |
| **Time from first PAH-related treatment to the index hospitalization admission date (days), mean ± SD** | 672.5 ± 513.4 | | 568.0 ± 555.1 | | 0.20 | |  | | 678.4 ± 556.3 | | | 0.01 |  |
| **Length of stay of index hospitalization (days), mean ± SD** | 8.6 ± 13.2 | | 10.6 ± 20.7 | | 0.11 | |  | | 8.8 ± 11.6 | | | 0.02 |  |
| **Time between discharge date and index date (days), mean ± SD** | 30.5 ± 25.3 | | 31.6 ± 24.6 | | 0.04 | |  | | 32.5 ± 24.2 | | | 0.08 |  |

† standardized difference >0.2

**Abbreviations:** ECG: electrocardiogram; PAH: pulmonary arterial hypertension; RHC: right heart catheterization; SD: standard deviation; STD: standardized difference.

## Table S3. Treatment patterns among weighted cohorts in the pre- and post-hospitalization period (sensitivity analysis among any treated patients during the pre-index hospitalization period)

| **PAH treatment** | **No treatment change cohort** | | **Escalation cohort** | | | |
| --- | --- | --- | --- | --- | --- | --- |
|  | **N = 2,023** | | **N = 315** | | | |
|  |  |  | **Pre** | | **Post** | |
| **Patients receiving PAH treatment** | 2,023 | (100.0) | 315 | (100.0) | 315 | (100.0) |
| Any PDE5i | 1,607 | (79.4) | 210 | (66.6) | 263 | (83.4) |
| Any ERA | 766 | (37.9) | 147 | (46.6) | 253 | (80.3) |
| Any sGCS | 98 | (4.8) | 23 | (7.2) | 43 | (13.6) |
| Any PPA | 537 | (26.5) | 47 | (14.8) | 204 | (64.9) |
| **Patients treated with monotherapy patientsSingle** | 1,252 | (61.9) | 212 | (67.2) | 0 | (0.0) |
| PDE5i | 950 | (47.0) | 129 | (40.9) | 0 | (0.0) |
| PPA | 150 | (7.4) | 17 | (5.3) | 0 | (0.0) |
| ERA | 132 | (6.5) | 58 | (18.5) | 0 | (0.0) |
| sGCS | 20 | (1.0) | 8 | (2.5) | 0 | (0.0) |
| **Patients treated with dual therapy** | 557 | (27.5) | 96 | (30.4) | 180 | (57.1) |
| PDE5i+ERA | 355 | (17.5) | 65 | (20.5) | 92 | (29.2) |
| PDE5i+PPA | 123 | (6.1) | 8 | (2.4) | 53 | (16.8) |
| ERA+PPA | 36 | (1.8) | 12 | (3.7) | 17 | (5.4) |
| ERA+sGCS | 29 | (1.4) | 5 | (1.5) | 11 | (3.6) |
| Other | 14 | (0.7) | 7 | (2.2) | 7 | (2.1) |
| **Patients treated with triple therapy** | 214 | (10.6) | 8 | (2.4) | 135 | (42.9) |
| PDE5i+ERA+PPA | 179 | (8.8) | 5 | (1.5) | 108 | (34.2) |
| ERA+sGCS+PPA | 35 | (1.7) | 3 | (0.9) | 17 | (5.3) |
| Other | 0 | (0.0) | 0 | (0.0) | 10 | (3.3) |

**Abbreviations:** ERA: endothelin receptor antagonist; PAH: pulmonary arterial hypertension; PDE5i: phosphodiesterase type 5 inhibitor; PPA: prostacyclin pathway agent; sGCS: soluble guanylate cyclase stimulator.

## Table S4. Primary diagnoses associated with rehospitalization among weighted cohorts (main analysis among patients treated with monotherapy during the pre-index hospitalization period)

| **Diagnosis (top 20 most frequent)** | **Monotherapy cohort**  **N = 1,252** | | **Escalation to combination therapy cohort (after weighting)**  **N = 203** | |
| --- | --- | --- | --- | --- |
| Shortness of breath | 200 | (16.0) | 24 | (11.7) |
| Pulmonary hypertension, unspecified | 147 | (11.7) | 34 | (16.8) |
| Heart failure, unspecified | 150 | (12.0) | 11 | (5.5) |
| Other nonspecific abnormal finding of lung field | 107 | (8.5) | 20 | (10.0) |
| Hypertensive heart and chronic kidney disease with heart failure and stage 1 through stage 4 chronic kidney disease, or unspecified chronic kidney disease | 81 | (6.5) | 9 | (4.4) |
| Acute and chronic respiratory failure with hypoxia | 79 | (6.3) | 15 | (7.4) |
| Pleural effusion, not elsewhere classified | 78 | (6.2) | 13 | (6.6) |
| Primary pulmonary hypertension | 77 | (6.2) | 19 | (9.4) |
| Chest pain, unspecified | 77 | (6.2) | 15 | (7.2) |
| Dyspnea, unspecified | 76 | (6.1) | 18 | (8.7) |
| Acute kidney failure, unspecified | 75 | (6.0) | 10 | (4.8) |
| Acute respiratory failure with hypoxia | 71 | (5.7) | 13 | (6.2) |
| Abnormal electrocardiogram [ECG] | 68 | (5.4) | 12 | (5.9) |
| Encounter for adjustment and management of vascular access device | 63 | (5.0) | 9 | (4.3) |
| Pneumonia, unspecified organism | 61 | (4.9) | 9 | (4.2) |
| Sepsis, unspecified organism | 61 | (4.9) | 3 | (1.5) |
| Hypertensive heart disease with heart failure | 60 | (4.8) | 9 | (4.5) |
| Unspecified atrial fibrillation | 60 | (4.8) | 6 | (2.8) |
| Cardiomegaly | 59 | (4.7) | 3 | (1.5) |
| Secondary pulmonary arterial hypertension | 46 | (3.7) | 13 | (6.6) |

## Table S5. Primary diagnoses associated with rehospitalization among weighted cohorts (sensitivity analysis among any treated patients during the pre-index hospitalization period)

| **Diagnosis (top 20 most frequent)** | | **No treatment change cohort**  **N = 2,023** | | **Escalation cohort**  **N = 315** | | |  |
| --- | --- | --- | --- | --- | --- | --- | --- |
| Shortness of breath | 309 | | (15.3) | | 52 | (16.6) | |
| Pulmonary hypertension, unspecified | 268 | | (13.2) | | 57 | (18.2) | |
| Heart failure, unspecified | 208 | | (10.3) | | 20 | (6.5) | |
| Other nonspecific abnormal finding of lung field | 165 | | (8.2) | | 21 | (6.8) | |
| Pleural effusion, not elsewhere classified | 123 | | (6.1) | | 15 | (4.7) | |
| Primary pulmonary hypertension | 153 | | (7.6) | | 29 | (9.3) | |
| Chest pain, unspecified | 129 | | (6.4) | | 23 | (7.3) | |
| Acute and chronic respiratory failure with hypoxia | 127 | | (6.3) | | 19 | (6.2) | |
| Secondary pulmonary arterial hypertension | 112 | | (5.5) | | 36 | (11.6) | |
| Dyspnea, unspecified | 118 | | (5.8) | | 23 | (7.4) | |
| Encounter for adjustment and management of vascular access device | 105 | | (5.2) | | 21 | (6.6) | |
| Acute respiratory failure with hypoxia | 104 | | (5.1) | | 20 | (6.4) | |
| Acute kidney failure, unspecified | 113 | | (5.6) | | 15 | (4.7) | |
| Cardiomegaly | 94 | | (4.6) | | 20 | (6.3) | |
| Sepsis, unspecified organism | 88 | | (4.3) | | 8 | (2.5) | |
| Unspecified atrial fibrillation | 78 | | (3.9) | | 9 | (2.8) | |
| Hypertensive heart and chronic kidney disease with heart failure and stage 1 through stage 4 chronic kidney disease, or unspecified chronic kidney disease | 102 | | (5.0) | | 9 | (2.7) | |
| Pneumonia, unspecified organism | 101 | | (5.0) | | 12 | (3.9) | |
| Abnormal electrocardiogram [ECG] | 95 | | (4.7) | | 16 | (5.0) | |
| Hypertensive heart disease with heart failure | 83 | | (4.1) | | 14 | (4.5) | |

## Figure S1. Hospitalization rates among weighted cohorts (sensitivity analysis among any treated patients during the pre-index hospitalization period)

**IRR (95% CI)**

**0.81 (0.66;0.99)**

***P*-value=0.040***

**Abbreviations:** CI: confidence interval; IRR: incidence rate ratio; PPPM: per-patient-per-month.
